# North by Southwest: Screening the Naturally Isolated Microalgal Strains from Different Habitats of Iran for Various Pharmaceutical and Biotechnology Applications

**DOI:** 10.1155/2022/4386268

**Published:** 2022-08-12

**Authors:** Zahra Dinpazhooh, Seyyed Vahid Niknezhad, Fardin Fadaei, Saeedeh Shaker, Ghasem Najafpour, Younes Ghasemi, Pegah Mousavi, Mohammad Hossein Morowvat

**Affiliations:** ^1^Pharmaceutical Sciences Research Center, Shiraz University of Medical Sciences, P.O. Box 71468-64685, Shiraz, Iran; ^2^Burn and Wound Healing Research Center, Shiraz University of Medical Science, P.O. Box 71987-54361, Shiraz, Iran; ^3^Department of Pharmaceutical Biotechnology, School of Pharmacy, Shiraz University of Medical Sciences, P.O. Box 71468-64685, Shiraz, Iran; ^4^Department of Chemical Engineering, Faculty of Engineering, Noshirvani University of Technology, Babol, Iran; ^5^Biotechnology Research Center, Shiraz University of Medical Sciences, P.O. Box 71468-64685, Shiraz, Iran; ^6^Department of Medical Genetics, Faculty of Medicine, Hormozgan University of Medical Sciences, Bandar Abbas, Iran

## Abstract

**Background and Aims:**

Microalgae are known as a promising source for food, pharmaceutical, and biofuel production while providing environmental advantages. The present study evaluates some newly isolated microalgal strains from north and southwest of Iran as a potential source for high-value products.

**Methods:**

Primitive screening was carried out regarding growth parameters. The molecular and morphological identifications of the selected strains were performed using 18S rRNA gene sequencing. After phylogenic and evolutionary studies, the selected microalgal strains were characterized in terms of protein and pigment content, in addition to the fatty acid profile content. Besides, the CO_2_ fixation rate was determined to assess capability for various environmental applications.

**Results:**

All of the selected strains were predominantly belonging to *Scenedesmus* sp. and *Desmodesmus* sp. The isolated *Scenedesmus* sp. VN 009 possessed the highest productivity content and CO_2_ fixation rate of 0.054 g·L^−1^d^−1^ and 0.1 g·L^−1^d^−1^, respectively. Moreover, data from GC/MS analysis demonstrated the high robustness of this strain to produce several valuable fatty acids including *α*-linolenic acid and linoleic acid in 45% and 20% of total fatty acids.

**Conclusions:**

The identified strains have a great but different potential for SCP, *β*-carotene, and *ω*-3 production, as well as CO_2_ fixation for environmental purposes. In this study, considering the wide range of microalgal strains in different habitats of Iran, the potential applications of native microalgae for various pharmaceutical, food, and biotechnology purposes were investigated.

## 1. Introduction

Nowadays, there is an increased affinity to use microalgae for a variety of industrial purposes including food, pharmaceutical, and biofuel production. Thanks to its renewability and being environmentally friendly, microalgae are considered as a robust candidate for CO_2_ bio-fixation, and also bioremediation of wastewater streams [1–4]. Biofuel from microalgae gained significant attention as an alternative fuel source, however, biofuel production from microalgae is faced with some major challenges since they are not economically sustainable. On the other hand, several successful attempts have been conducted toward exploiting microalgae as a food and feed source or other pharmaceutical biotechnology applications [5–7].

Microalgae possess a range of advantages including high lipid content, high growth rate; the ability to grow in poor quality waters and on non-arable lands while; at the same time, providing environmental benefits [8–10].

Microalgae have demonstrated diverse adaptation in natural or man-made ecosystems such as fresh, saline water, wastewater, and soil. Microalgae widely occurred in freshwater and marine habitats such as lakes, rivers, ponds, and streams [11]. Algae have been estimated over 1 million species around the world according to AlgaeBase. Currently, about 165,000 species have been processed by AlgaeBase (https://www.algaebase.org) to date [12]. Therefore, exploiting indigenous strains that possess high dominance and adaptability to local environmental conditions should be a rational strategy to obtain novel isolates with potential applications to produce bioactive compounds.

A vast range of energy and non-energy product can be obtained from microalgae based on their potential [13]. Hence, the only biodiesel or single production line approach cannot be commercially viable compared to the matrix approach. To date, the system of biomass Biorefining led to the sustainable processing of biomass into a wide spectrum of marketable products [14, 15]. The various co-products derived from the algal biorefinery like lipids, proteins, carbohydrates, polyunsaturated fatty acids (PUFAs), pigments, natural colorants, antioxidants, as well as pharmaceutical active ingredients can be produced from microalgal biomass. In addition to the biorefinery approach, nowadays, combinatorial genetic engineering based on increasing the number of the gene of interest is being addressed toward more efficient biosynthesis and more industrial reality [13].

Microalgae is an emerging alternative protein source that could meet expected global protein requirements in food and feed applications. Environmental problems that are associated with the high ecological footprint of conventional agriculture practices resuscitated the interest in microalgae as a source of sustainable protein [16–18]. Freshwater strains can produce high amounts of protein up to 70% DW, which is composed of essential amino acids that are equal to or even superior compared to conventional plant sources. High quality amino acids, environmentally friendly feathers like CO2 consumption, and not needing arable land or drinking water have made microalgae as alternative food source. Nowadays*, Spirulina* and *Chlorella* have been known as rich sources of protein content (about 60%) as well as mineral, carotenoid, antioxidant, and vitamin. *Spirulina* is being incorporated into many foods' formulations including bread, pasta, and dairy product, most of which, use as coloring agents or as a marketing strategy. Both *Spirulina* and *Chlorella* show very similar benefits in terms of the bioactive compounds that affect our physiology. *Chlorella* is higher in fat and calories when compared to *spirulina* while containing higher amount of minerals and vitamins. While *Spirulina* may have 10 percent more protein. This algal biomass has positioned firmly in the food and beverage, nutraceutical, and pharmaceutical market [18–21]. However, the industry has faced some critical challenges including undesirable flavor, color, and eventually consumer acceptance. To date, the major microalgal market can be found in the health food market in the form of encapsulated algal powder. The suitability of these microalgae as a feed supplement, mainly for Poultry and aquaculture is demonstrated by a large number of nutritional and toxicological evaluations [19, 20].

Currently, single-cell protein (SCP) products from microalgae and microbial sources are actively being commercialized as a food ingredient in aquaculture [22, 23].

Microalgal species are also recognized as the richest source of pigments, mainly including chlorophyll *a*, *b*, and *c*; *ß*-carotene, xanthophylls, phycocyanin, and phycoerythrin. Carotenoid pigments are widely used in food, pharmaceuticals, and cosmetics. Microalgal carotenoid has been indicated various health benefits; it is converted to vitamin A in the human body. which is assist body's immune system and also has antioxidant property that eliminates the harmful effects of free radicals which are implied several diseases such as premature aging, various forms of cancer, coronary heart disease, and arthritis [21]. Nowadays, *ß*-carotene has been widely commercially applied as natural food and cosmetic colorant because of an increasing global trend and consumer preference toward natural products [24]. Commercially viable carotenoids are extracted from *Dunaliella salina* yielding up to 400 mg *ß*-carotene m^−2^ of cultivation area under ideal conditions [25].

Algal lipid content could be increased up to 75% DW by nitrogen depletion [26–28], genetic modifications [29], and exploring novel microalgae strains. Although microalgal lipid has mainly been considered as a feed for biodiesel production, still not economically competitive compared to terrestrial plants and lacks an evaluation of its full potential [30]. Recently obtaining high-value compounds from algal lipids, especially polyunsaturated fatty acids (PUFA), has aroused much more attention in the food and pharmaceutical industries [31–33]. The polyunsaturated fatty acid comprises some major fatty acids, such as essential fatty acids *ω*-3, *ω*-6, and conjugated fatty acids [34]. Docosahexaenoic acid (DHA; 22 : 6 *n* − 3) and eicosapentaenoic acid (EPA; 20 : 5 *n* − 3) along with *α*-linolenic acid (ALA; 18 : 3 *n* − 3) and docosapentaenoic acid (22 : 5 *n* − 3) known as essential fatty acids (EFAs), which are imperative for human health [35]. DHA possesses a cardioprotective function; as a consequence, it is commonly used as a nutraceutical agent in nutritional products such as infant formula, dairy, and certain other food categories [21, 36]. There are extensive studies using the strain of *Schizochytrium* sp. and *Crypthecodinium cohnii* for scale-up production of DHA [37–39].

Owing to its climate diversity as well as a variety of natural resources, Iran has been considered as one of the excellent sources to find a large number of unstudied microalgal strains with special metabolic abilities [34, 40]. However, there has not been a comprehensive study on the screening of naturally isolated microalgae to assess high-value products, a possibility for large-scale production. Hence, this study was conducted to investigate naturally isolated microalgal strains from some habitats close to the Caspian Sea (north of Iran) and Kohgiluyeh and Boyer-Ahmad provinces (southwest of Iran), as precious reservoirs for different microalgal strains, to identify the best strain regarding biomass productivity, biochemical compositions along with providing the environmental benefits, to assess information for industrial application of high-value products.

## 2. Materials and Methods

### 2.1. Isolation and Cultivation

The microalgal strains used in this study were aseptically isolated, from various habitats including rivers, headwaters, paddy fields, and a sea, which are located in Kohgiluyeh and Boyer-Ahmad provinces (30 40′ 12″ N, 51 36′ 0″ E) and Mazandaran province (36° 42′ 9″ N, 52° 39′ 27″ E), the southwest and north of Iran, respectively. Water and wet soil samples were taken from the water surface and up to 5 cm of the top of the soil.

**Figure 1:**
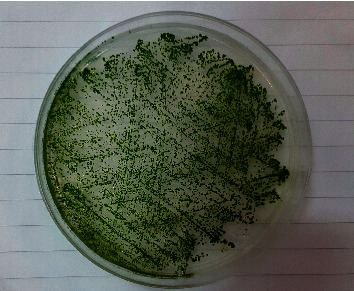
An agar plate streaked showing small isolated colonies arising from a single cell.

Preliminary cultivation and isolation of the microalgal strains were performed in Pharmaceutical Sciences Research Center, Shiraz University of Medical Sciences. The collected samples were enriched using BG-11 liquid medium described in Table 1 [41]. They were incubated under constant illumination at 60 mol·m^−2^·s^−1^ intensity with 12 : 12 h dark: light photoperiod of white fluorescent lamps at 25 ± 2°C for three weeks. After a visible growth occurred, the purified colonies were obtained by repeated sub-culturing cells across BG-11 solid agar plates and regular microscopic observation (Figure 1). The pure unialgal colonies were inoculated into a new 20 mL flask containing liquid BG-11 medium, then incubated in the abovementioned culture room.

### 2.2. Morphological Identification

**Figure 2:**
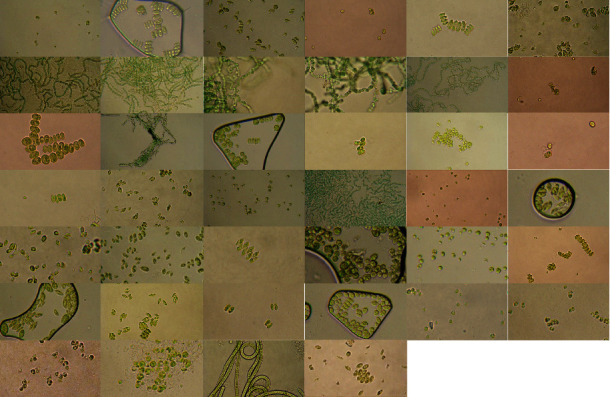
Light microscopy photograph of all isolated microalgal strains using a 40*X* microspore lens.

Preliminary identification of isolated microalgae was performed by direct samples microscopy of BG-11 culture (10 *μ*L) between 10 to 14 days. Among forty isolated microalgae (Figure 2), a total of twenty-nine single-cell strains were identified by evaluation of morphologic traits (cell shape, size, chloroplast, pyrenoid, flagellum, and cells arrangement) using an Olympus BX41 optical microscope equipped with an Olympus DP80 CCD camera, based on standard morphological feature keys [42, 43].

### 2.3. Molecular Identification of Selected Microalgae

#### 2.3.1. Primers and PCR Condition

A polymerase chain reaction (PCR) targeting 18S rRNA was used to identify the isolated microalgae. Genomic DNA was extracted using a DNP™ DNA extraction kit (SinaClon, Tehran, Iran) according to the manufacturer's instructions and adjusted to the final concentration of 20 ng·*μ*L^−1^.

The partial 18S rRNA gene was PCR amplified using a universal primer set; 5′-GTCAGAGGTGAAATTCTTGGATTTA-3′ (Forward), and 5′-AGGGCAGGGACGTAATCAACG-3′ (Reverse) [40] in a total reaction volume of 80 *μ*L containing: 40 *μ*L Taq DNA polymerase master mix red 2X (Ampliqon, Odense, Denmark), 3 *μ*L of each primer (20 *μ*M), 6 *μ*L template DNA and 28 *μ*L sterile deionized distilled water. The PCR conditions were as follows: initial denaturation at 95°C for 5 min, followed by 30 cycles of denaturation at 95°C for 1 min, annealing at 58°C for 1 min, elongation at 72°C for 90 s, and the final extension step at 72°C for 90 s, according to the method used by Radha et al. [44] with slight modifications.

#### 2.3.2. Electrophoresis and Gel Purification

The 1.8% Tris-Borate-EDTA (TBE) agarose gel and U: Genius3 GelDoc system (SYNGENE, Cambridge, United Kingdom) was used for electrophoresis and visualization of PCR products. The desired amplicons (∼750 bp) were extracted and purified using a Gel Purification Kit (Bioneer, Daejeon, South Korea) and sent for sequencing (Macrogen, Seoul, South Korea).

### 2.4. Analyzing Sequences

#### 2.4.1. Multiple Sequence Alignment

The BioEdit (version 7.1.9) and Chromas Pro (version 2.1.3) programs were utilized to modify the obtained sequences. The final resulting 18S rRNA gene sequence was analyzed and used the BLASTn search tool for finding similarity searches against previously published 18S rRNA gene sequences in the NCBI databases. The representative sequences were selected and aligned using CLC Genomics workbench V20.0.

#### 2.4.2. Molecular Phylogeny

A phylogenetic tree was constructed with the Neighbor-Joining algorithm using CLC genomics software version 20.0 with a bootstrap analysis of 500 replicates. The 18S rRNA gene sequence obtained in this study was deposited in GenBank databases.

### 2.5. Screening of Isolated Microalgae for their Growth Potential

#### 2.5.1. Cell Dry Weight Measurement

A total of twenty-nine isolated strains were inoculated in 20 mL flasks (10% (v/v)) containing 12 mL of BG-11 medium and were kept on a rotary shaker at 150 rpm and 20°С under a 12 : 12 h LD cycle of 100 *μ*E·m^−2^·S^−1^ illumination.

The experiment was performed for 18 days. The sampling procedure was exploited every two days. The biomass concentrations were assessed gravimetrically. Biomass was harvested at 10000 rpm, for 5 min at 4°C and washed twice with distilled water followed by drying at 60°C in an oven for 48 h. Eventually, ten candidate microalgae were selected from the above step based on their high biomass productivity for further studies.

#### 2.5.2. Growth Measurement and Kinetics

The biomass productivity (BP, gL^−1^·day^−1^), maximum specific growth rate (*μ*_max_ day^−1^), and doubling time (h) were determined using equations (1), (2), and (3), respectively:

(1) $$\begin{array}{c} \text{BP}=\frac{X2-X1}{t2-t1}, \end{array}$$

(2) $$\begin{array}{c} \mu_{\max}=\frac{\ln\;X2-\ln\;X1}{t2-t1}, \end{array}$$

(3) $$\begin{array}{c} t_{d}=\frac{\ln\;2}{µ\max}, \end{array}$$

where *X*_1_ and *X*_2_ represent the dry cell weight (DCW, gL^−1^) and (*t*_2_ − t_1_) is required time for increasing cell concentration from *X*_1_ to *X*_2_ in the exponential phase [45]. All the experiments were done in triplicates to calculate standard deviations.

### 2.6. Biochemical Analysis of Selected Strains

#### 2.6.1. Protein Content Analysis

Total protein contents were evaluated by Bradford method. A calibration curve was assessed using bovine serum albumin. Freeze-dried biomass of each sample (5 mg) that was harvested on the 18th day, was assayed for alkaline hydrolysis in 1 mL NaOH (1 N) for 1 h at 100°C using a hot plate (WiseTherm HB-R) and then centrifuged at 10000 rpm for 3 min. Protein content of each sample was calculated by measuring absorbance and comparing their absorbance at 595 nm with the bovine serum albumin standard curve [40]. This analysis was performed in triplicates and the average values were used for statistical evaluation of the results.

#### 2.6.2. Pigments Analysis

*(1) Determination of Chlorophyll a, Chlorophyll b, and Total Chlorophyll*. Chlorophyll concentration determination was defined according to the method described by Eijckelhoff and Dekker [46]. Three mL of the microalgae suspension was taken and centrifuged at 2500 rpm for 10 min and rinsed with acetone (80%) and centrifuged again. The definition of the amount of chlorophyll extracted in the mentioned method was determined by colorimetric assay at 668.2 and 646.2 nm using an ELISA plate reader with the following equation:

(4) $$\begin{array}{c} \text{Chlorophyll }a\left(\frac{\mu \text{g}}{\text{mL}}\right)=12.25\times A_{668.2}-2.79\times A_{646.2}. \end{array}$$

Five mL of the microalgae suspension was taken and added to the 3 mL acetone (80%), and the definition of the amount of total chlorophyll (*a* + *b*) extracted was specified using the following equation:

(5) $$\begin{array}{c} \text{Total Chlorophyll}a\left(a+b\right)\left(\frac{\mu \text{g}}{\text{mL}}\right)=7.93\times A_{664}-19.53\times A_{647}. \end{array}$$

*(2) β-Carotene Extraction and Assay*. β-carotene extraction from the cell pellets was performed using the ethanol/n-hexane (2 : 1, v/v). The microalgal suspension (1 mL) was collected, centrifuged at 3000 rpm for 5 min, and rinsed with ethanol/*n*-hexane (2/1) and distilled water. *β*-carotene concentration is solved in the overlapping phase with n-hexane was determined by colorimetric assay at 450 nm using ELISA plate reader [47]. The definition of the amount of *β*-carotene extracted in n-hexane was specified using the following equation:

(6) $$\begin{array}{c} \beta -\text{carotene}\left(\frac{\mu \text{g}}{\text{mL}}\right)=25.2\times A_{450}. \end{array}$$

### 2.7. CO_2_ Fixation Rate

By removing the carbon source from BG-11 media (Na_2_CO_3_), CO_2_ fixation rate for each selected strain was calculated in terms of CO_2_ consumption by each selected strain according to the following equation:

(7) $$\begin{array}{c} R_{\text{CO}2}\left({\text{mgL}}^{-1}{\text{d}}^{-1}\right)=P\times C_{\text{carbon}}\times \left(\frac{M_{\text{CO}2}}{M_{C}}\right), \end{array}$$

where *R*_CO2_ is the rate of CO_2_ fixation (mg·L^−1^·d^−1^), *P* is the biomass productivity in 18 days (mg·L^−1^·d^−1^), *M*_CO2_ and *M*_*C*_ represent the molecular weight of carbon dioxide and atomic weight of carbon, respectively. *C*_carbon_ is the carbon content of the biomass was measured by a CHNS-O elemental analyzer equipped with gas chromatography and TCD detector (Costech, ECS 4010) [48].

### 2.8. FAME Analysis

#### 2.8.1. Lipid Extraction

Fatty acid extraction was performed according to the Bligh and Dyer method with some modifications [49]. Briefly, lyophilized microalgal biomass (1 g) was boiled in 5 mL of isopropanol for 2 min and dried by vacuum rotary. A mixture of and 5 mL of chloroform was added, followed by 5 mL of potassium chloride solution (0.88%), to give a final solvent ratio of chloroform: methanol: water of 1 : 1 : 0.8. The mixture chloroform-methanol (1 : 2 v/v) was added to dried pellet in the presence of 0.015 g butylated hydroxytoluene (BHT) as an antioxidant agent, vortexed for 30 s and centrifuged (2000*g*, 5 min). To the above-obtained supernatant, 0.8 mL distilled water was shaken for 5 min using a separating funnel and allowed for 30 min to be separated. The total lipid was collected by concentrating the solvent phase under nitrogen gas.

#### 2.8.2. Fatty Acid Esterification and GC/MS Analysis

The acid-catalyzed esterification was carried out in the Dien–Stark apparatus and was heated to reflux for 24 h at 75°C. The reaction mixture consisted of 0.5 g of extracted fatty acid dissolved in 3 mL methanol in the presence of 0.3 mL sulfuric acid. The mixture was washed with 4 mL saturated sodium hydrogen carbonate and then dried over anhydrous sodium sulfate. To assess an oily substance ready for GC/MS analysis, the solvent was removed under nitrogen gas [40].

The GC/MS analysis was performed using an Agilent 7890A GC 5977B MSD equipped with an HP-5MS capillary column (30 m × 250 *μ*m × 0.25 *μ*m, Agilent 19091S-433). The oven temperature was programmed between 70°C (5 min) to 270°C for 10 min and the rate was 7°C·min^−1^. The carrier gas was helium with a flow rate of 1 mL·min^−1^. The identification of fatty acid peaks was carried out by comparing the obtained mass spectra with the Wiley 7n.1 library.

## 3. Results and Discussion

### 3.1. Isolation and Identification of Microalgal Strains

A total of forty microalgal strains were isolated from thirteen samples collected along the southern coast of the Caspian Sea, Mazandaran province, and different habitats of Kohgiluyeh and Boyer-Ahmad provinces, the southwest of Iran. To obtain the biodiversity of mentioned regions in the south and north of the country, samples were collected from different habitats of these locations.

Microscopic observation of these isolated microalgae indicated their colonial existence and purities which includes unicellular, colonial, and filamentous forms of Chlorophyceae and Cyanophyceae families. Photomicrograph of all isolated strains is presented in Figure 2. The bio-diversity of strains is quite likely that reflects the vast adaptation ability of these strains in the freshwaters.

All isolated microalgae were represented with specific codes and maintained in Microalgal Culture Collection of Shiraz University of Medical Sciences. Twenty-nine single-cell isolated strains were screened. Among them, ten strains were selected based on their high biomass productivity, and subjected to more characterizations. Preliminary morphology-based identification showed that the most morphological features of these algal isolates resemble the genus of *Scenedesmus*, which belongs to the family of Scenedesmaceae within the class of Chlorophyceae. Photomicrographs and morphological features of ten selected algal isolates are shown in supplementary data, Figure S1 and Table S1, respectively.

Metzger and Largeau noted that for the same strain and within each chemical race, the morphological feature could vary regarding age and culture conditions [50]; therefore, PCR-amplification of 18S rRNA was used to confirm our morphological identification.

### 3.2. PCR Amplification and Phylogenetic Analysis

**Figure 3:**
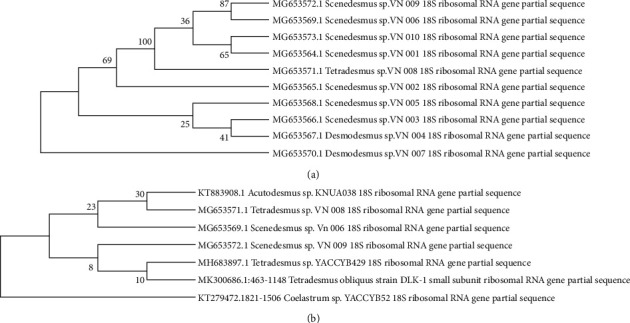
(a) Phylogenic analysis of ten microalgal isolates with higher biomass productivities using MEGA-X analyzed by maximum likelihood (distance measure = Jukes-Cantor model, bootstrap = 500 replicates) with nearest neighbor interchange. (b) Phylogenic tree of three promising strains (*Scenedesmus* sp. VN009, *Scenedesmus* sp. VN006, and *Tetradesmus* sp. VN008) by compared with other closely related species using MEGA-X analyzed by maximum likelihood (distance measure = Jukes-Cantor model, bootstrap = 500 replicates) with nearest neighbor interchange.

Molecular identification of ten selected microalgae, which displayed high productivity, was performed based on the partial 18S rRNA gene by comparing them with the reference sequences retrieved from the GenBank database. All of the selected strains belonged to the Chlorophyceae including, the genera *Tetradesmus, Desmodesmus, and Scenedesmus* as the most abundant genus among them. Accession numbers of these sequences from GenBank and also the relationships among partial 18S rRNA that was inferred using phylogenic analysis by Neighbor-Joining algorithm are indicated in Table 2 and Figure 3(a), respectively. In parallel, the phylogenetic analysis was represented and compared with *Acutodesmus* sp., *Coelastrum* sp., and *Tetradesmus* sp. as similar species (Figure 3(b)).

### 3.3. Kinetics and Growth Parameters

**Figure 4:**
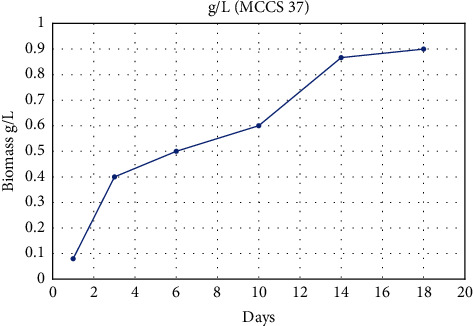
Growth curve of typical isolated microalgal strain (MCCS 37) from Kohgiluyeh and Boyer-Ahmad provinces.

A total of twenty-nine single-cell strains were cultured in BG-11 under uniform conditions as described in Section 2.5.1 section. During this period, the growth curve of one typical isolated strain was portrayed in Figure 4. All cultures were harvested at the end of the exponential phase of the typical isolate (18th day).

Screening of the isolated microalgae has been performed primarily by growth characterization analyses, Table S2. After the screening program, ten strains were chosen where the biomass productivity was the main selection parameter and identified afterward (Table 3).

The observed biomass productivities were revealed to be higher than average values for previously reported concentrations, 0.03 gL^−1^·d^−1^ [11], 0.016 gL^−1^·d^−1^ [51]. The highest productivity among these selected strains belongs to *Scenedesmus* sp. VN009 (MCCS35), reaching 0.054 gL^−1^·d^−1^, followed by *Scenedesmus* sp. VN006 (MCCS25) and *Tetradesmus* sp. VN008 (MCCS32) at 0.044 gL^−1^·d^−1^. The lowest biomass productivity was found in two strains of *Desmodesmus* (*Desmodesmus* sp. VN007: 0.032 gL^−1^·d^−1^; *Desmodesmus* sp. VN004: 0.035 gL^−1^·d^−1^).

These findings warrant the high potential of our selected strains to be exploited for scale-up studies for SCP production, or food and biofuel.

### 3.4. Protein and Pigment Content of the Selected Isolated Microalgae

The quantitative analysis of protein and pigment of the ten chosen strains is shown in Table 4. The isolated *Desmodesmus* sp. VN 004 and *Scenedesmus* sp. VN 010 strains showed the highest protein content among the ten strains assayed (*Desmodesmus* sp. VN 004: 21.50% DW; *Scenedesmus* sp. VN 010: 19.30% DW). *Scenedesmus* sp. VN 006 protein content (18.58% DW) was similar to that of *Scenedesmus* sp. VN010 (Table 3). The least observed amount of protein was comprehended in *Scenedesmus* sp. VN 001 with 12.10% DW. It should be mentioned that the provided data in Table 4 only account for a small fraction of the weight percent as protein. The other identified parts were lipids, carbohydrates, nucleic acid fractions, other impurities, and possible errors.

The typical amount of protein in different microalgae during physiologic conditions has been reported to be about 10% to 71% DW [20]. However, the composition of culture medium and seasonal change might have influenced biomass content and compositions. Besides, the natural specifications of each strain are crucial as well.

The *ß*-carotene content of all the ten selected microalgae indicated that the maximum value of *ß*-carotene content (0.372% DW) was achieved by *Scenedesmus* sp. VN 010, followed by *Scenedesmus* sp. VN 001 (0.186% DW) and *Scenedesmus* sp. VN 003 (0.142% DW). The *ß*-carotene content in *Scenedesmus* sp. VN 010 was found to be significantly more than the second highest strains (about twice the amount). *ß*-carotene possesses anti-oxidant activity; therefore, it plays an important role in human health. It is usually present in the range of 0.1–0.2% of the total dry weight of microalgae and up to 14% DW in industrialized species [52]. Different approaches of bioprocess and genetic engineering seem to be useful for enhancing the *ß*-carotene content of isolated microalgae for different industrial applications.

Results from Table 4 also show the highest chlorophyll content is owing to *Scenedesmus* sp. VN 009 (516.74 *μ*moles).

### 3.5. CO_2_ Fixation Rate

To avoid the role of the carbon source of BG-11 culture medium in CO_2_ fixation by selected microalgae, the main source of culture medium (Na_2_CO_3_) was removed, and the ability to stabilize CO_2_ in the air was investigated. Concerning the CO_2_ fixation rate, the data shown in Table 5 reveal that *Scenedesmus* sp. VN 009 had the best performance of 0.101 gL^−1^·d^−1^. The rate of carbon dioxide photosynthetically fixed by the rest of the strains was almost similar ranging from 0.067 to 0.078 gL^−1^·d^−1^, except for *Desmodesmus* sp. VN 007 that showed the lowest CO_2_ fixation rate of 0.042 gL^−1^·d^−1^. The maximum content of the CO_2_ fixation rate belonged to the strain, which was isolated from around Babol with −2 up to 50 m elevations, in contrast, those strains have been isolated from Yasuj with an average height of 1810 m height. It would seem that the strain isolated from the region with less height shows more CO_2_ fixation adaptability.

Previous reports indicate that *Scenedesmus* spp. Strains are drawn extensive attention for their appropriate CO_2_ fixation ability. Similarly, Isolated strains in this study have exhibited excellent performance in CO_2_ fixation, and the results are quite comparable to previous research, which have CO_2_ fixation rate in the range of 0.03 to 0.06 gL^−1^·d^−1^ with regard to their control conditions with 0.03% CO_2_ (v/v) [53–55].

Considering the recent environmental issues such as global warming, industrialization, and population growth, having renewable energy and discovering potential strains for CO_2_ fixation are vitally important.

According to obtained results for CO_2_ fixation, it could be suggested that isolated microalgae in this study are quite appropriate for CO_2_ fixation purposes.

### 3.6. Fatty Acid Composition

The Fatty acid profile gives vital information to choose the strain with the targeted application. Hence, Table 6 is prepared to show the fatty acid profile of the top three isolated microalgae based on high biomass productivity values.

The results of GC/MS analysis comprehended that the three selected microalgal strains contain valuable saturated fatty acids (SFA). Palmitic acid (C 16 : 0), Stearic acid (C 18 : 0), and Arachidic acid (C 20 : 0) were regarded as the major identified SFAs. It can be observed that the algal lipid comprised high content of PUFA, ranging from 43% to 80%, which is desirable for pharmaceuticals and food industries.

Linoleic acid (*ω*-6), *α*-linolenic acid (*ω*-3), and *ɤ*-linolenic acid (*ω*-6) (only in *Scenedesmus* sp. VN 009) are considered as the valuable PUFAs. *Scenedesmus* sp. VN 009 exhibited 45% *ω*-3 production, which was higher than previously reported [11, 56, 57]. This indicates a great promising candidate for *ω*-3 production. Also, *Scenedesmus* sp. VN 006 *α*-linolenic acid content was almost similar to that of *Scenedesmus* sp. VN009 (35% of total fatty acid). These polyunsaturated fatty acids have been known as essential precursors of longer n-3 PUFAs, especially DHA, that eventually could possess several beneficial impacts on human health, from influencing fetal growth and development to reducing body fat [58], prevention of cardiac arrhythmias, sudden cardiac death [59], Alzheimer's disease [33], alleviate coronary heart disease [60], and also diabetes [61].

Furthermore, there are some studies indicate that *α*-linolenic acid (ALA) and linoleic acid have exerted therapeutic effects as they could reduce ischemic brain damage, and alleviate functional stroke recovery and psychiatric disorders [62]. Furthermore, details are presented in Table 6.

## 4. Conclusions

To sum up, we have isolated twenty-nine microalgal strains from different locations in Iran, selected strains were further characterized to find the most promising strains for food along with CO_2_ fixation ability. *Scenedesmus* sp. and *Desmodesmus* sp. were the most abundant microalgae in the Kohgiluyeh and Boyer-Ahmad provinces (southwest of Iran). Some strains showed high biomass productivity and CO_2_ fixation rate. Fatty acid analyzed in this study shows the ability of *Scenedesmus* sp. VN009 and *Scenedesmus* sp. VN 006 to accumulate a high level of *α*-linolenic acid (*ω*-3). As far as we know, the observed amounts for *ω*-3 productions were higher than any other available reports in the literature, thereby these strains could be targeted for large-scale production.

## Figures and Tables

**Table 1:** BG-11 culture medium composition.

Compound	Amount (g/L)
NaNO_3_	1.5
K_2_HPO_4_	0.04
MgSO_4_.7H_2_O	0.075
CaCl_2_.2H_2_O	0.036
Citric acid	0.006
Ferric ammonium citrate	0.006
EDTA	0.001
Na_2_CO_3_	0.002
Trace-elements solution^*∗*^	1mL/L

^*∗*^H_3_BO_3_, 2.86 g/L; MnCl_2_.4H_2_0, 1.81 g/L; ZnSO_4_.7H_2_0, 0.222 g/L; Na_2_MoO_4_.2H_2_O, 0.39 g/L; CuSO_4_.5H_2_O, 0.079 g/L; Co (NO3)_2_ .6H_2_O, 0.0494 g/L.

**Table 2:** Accession numbers for 18S rRNA gene sequence, strain, family, length in base pairs, and location for ten microalgal isolates.

Code	Accession number	Strain	Family	Length (bp)	Location
MCCS^*∗*^1	MG653564.1	*Scenedesmus* sp. VN 001	Chlorophyceae	567	Yasuj, 25 Km SE
MCCS4	MG653565.1	*Scenedesmus* sp. VN 002	Chlorophyceae	596	Yasuj, 30 Km SE
MCCS17	MG653566.1	*Scenedesmus* sp. VN 003	Chlorophyceae	687	Yasuj, 12 Km NW
MCCS20	MG653567.1	*Desmodesmus* sp. VN 004	Chlorophyceae	671	Yasuj, 25 Km NW
MCCS21	MG653568.1	*Scenedesmus* sp. VN 005	Chlorophyceae	623	Yasuj, 25 Km NW
MCCS25	MG653569.1	*Scenedesmus* sp. VN 006	Chlorophyceae	701	Yasuj, 30 Km SE
MCCS28	MG653570.1	*Desmodesmus* sp. VN 007	Chlorophyceae	224	Yasuj, Beshar river SE
MCCS32	MG653571.1	*Tetradesmus* sp. VN 008	Chlorophyceae	613	Yasuj, 25 Km NW
MCCS35	MG653572.1	*Scenedesmus* sp. VN 009	Chlorophyceae	686	Babol, 70 Km·S
MCCS41	MG653573.1	*Scenedesmus* sp. VN 010	Chlorophyceae	688	Yasuj, 125 Km SW

^*∗*^Microalgal culture collection of Shiraz university of medical sciences (MCCS).

**Table 3:** Top ten biomass productivity values of the isolated microalgae.

Strain	Specific growth rate (d^−1^)	Doubling time (day)	Biomass yield (gL^−1^)	Biomass productivity (gL^−1^·d^−1^)
*Scenedesmus* sp. VN 009	0.085	8.12	0.97 ± 0.10	0.054 ± 0.005
*Scenedesmus* sp. VN 006	0.073	9.45	0.79 ± 0.09	0.044 ± 0.005
*Tetradesmus* sp. VN 008	0.089	7.75	0.80 ± 0.10	0.044 ± 0.005
*Scenedesmus* sp. VN 010	0.084	8.21	0.74 ± 0.08	0.041 ± 0.004
*Scenedesmus* sp. VN 001	0.079	8.73	0.72 ± 0.03	0.040 ± 0.001
*Scenedesmus* sp. VN 002	0.08	8.62	0.74 ± 0.09	0.040 ± 0.005
*Scenedesmus* sp. VN 003	0.096	7.19	0.70 ± 0.07	0.039 ± 0.004
*Scenedesmus* sp. VN 005	0.085	8.12	0.68 ± 0.07	0.038 ± 0.004
*Desmodesmus* sp. VN 004	0.072	9.58	0.63 ± 0.07	0.035 ± 0.004
*Desmodesmus* sp. VN 007	0.064	10.78	0.58 ± 0.09	0.032 ± 0.005

**Table 4:** Biomass composition analysis of selected microalgal strains.

Strain	Protein (% w/w)	Total *β*-carotene (% w/w)	Total *β*-carotene (*μ*g·mL^−1^)	Chlorophyll *a*(*μ* moles)	Chlorophyll *b*(*μ* moles)	Total chlorophyll (*μ* moles)
*Scenedesmus* sp. VN 001	12.10 ± 1.50	0.186 ± 0.13	1.77 ± 0.14	60.08 ± 0.29	269.13 ± 0.52	343.82 ± 0.94
*Scenedesmus* sp. VN 002	14.35 ± 1.90	0.036 ± 0.09	0.35 ± 0.07	237.53 ± 0.11	172.27 ± 0.39	423.1 ± 0.88
*Scenedesmus* sp. VN 003	16.40 ± 1.10	0.142 ± 0.12	1.21 ± 0.24	139.25 ± 0.43	247.60 ± 0.70	401.95 ± 0.73
*Desmodesmus* sp. VN 004	21.50 ± 3.12	0.04 ± 0.01	0.35 ± 0.10	7.54 ± 0.11	278.04 ± 0.81	299.58 ± 0.89
*Scenedesmus* sp. VN 005	17.12 ± 0.51	0.052 ± 0.010	0.45 ± 0.06	13.2 ± 0.08	231.62 ± 0.74	256.62 ± 0.92
*Scenedesmus* sp. VN 006	18.58 ± 5.08	0.075 ± 0.021	0.81 ± 01	59.26 ± 0.29	337.72 ± 0.97	414.98 ± 1.07
*Desmodesmus* sp. VN 007	12.75 ± 1.36	0.047 ± 0.005	0.4 ± 0.0	19.79 ± 0.37	228.99 ± 0.73	260.58 ± 0.99
*Tetradesmus* sp. VN 008	12.75 ± 3.00	N. d^*∗*^	N. d	67.41 ± 0.22	250.15 ± 0.66	331.36 ± 1.21
*Scenedesmus* sp. VN 009	15.23 ± 2.12	0.057 ± 0.009	0.71 ± 0.03	165.43 ± 0.41	331.51 ± 0.85	516.74 ± 1.44
*Scenedesmus* sp. VN 010	19.30 ± 3.13	0.372 ± 0.056	3.53 ± 0.08	176.65 ± 0.25	136.32 ± 0.63	323.28 ± 1.01

^*∗*^Not determined.

**Table 5:** CO_2_ fixation rate was observed in each studied microalgal strain.

Strain	Carbon concentration in biomass	Biomass productivity (gL^−1^·d^−1^)	CO_2_ fixation rate (gL^−1^·d^−1^)
*Scenedesmus* sp. *VN 009*	50.90	0.054 ± 0.005	0.101 ± 0.009
*Scenedesmus* sp. *VN 006*	43.63	0.044 ± 0.005	0.071 ± 0.008
*Tetradesmus* sp. *VN 008*	48.18	0.044 ± 0.005	0.078 ± 0.008
*Scenedesmus* sp. *VN 010*	48.43	0.041 ± 0.004	0.073 ± 0.007
*Scenedesmus* sp. *VN 001*	53.17	0.040 ± 0.001	0.078 ± 0.002
*Scenedesmus* sp. *VN 002*	51.15	0.040 ± 0.005	0.075 ± 0.009
*Scenedesmus* sp. *VN 003*	51.90	0.039 ± 0.004	0.074 ± 0.008
*Scenedesmus* sp. *VN 005*	50.19	0.038 ± 0.004	0.070 ± 0.007
*Desmodesmus* sp. *VN 004*	51.94	0.035 ± 0.004	0.067 ± 0.008
*Desmodesmus* sp. *VN 007*	50.55	0.032 ± 0.005	0.042 ± 0.009

**Table 6:** Fatty acid composition profile for microalgal lipids from three isolates exhibiting the highest biomass productivities.

Fatty acids	*Scenedesmus* sp. VN 006	*Tetradesmus* sp. VN 008	*Scenedesmus* sp. VN 009
Myristic acid (C14 : 0)	—	0.69	0.639
Palmitic acid (C16 : 0)	47.23	9.05	21.13
Palmitoleic acid (C16 : 1) (*ω*-7)	—	0.74	—
7,10-Hexadecadienoic acid (C16 : 2)	—	0.72	—
Stearic acid (C18 : 0)	9.57	8.52	3.13
Oleic acid (18 : 1) (*ω*-9)	—	—	5.48
Linoleic acid (C18 : 2) (*ω*-6)	8.09	53.32	20.7
*α*-linolenic acid (C18 : 3) (*ω*-3)	35.09	26.93	45.43
*γ*-linolenic acid (C18 : 3) (*ω*-6)	—	—	1.21
Arachidic acid (C20 : 0)	—	—	0.63
Behenic acid (C22 : 0)	—	—	0.67
Lignoceric acid (C24 : 0)	—	—	0.94
PUFA	43.18	80.97	67.34

## Data Availability

All generated or analyzed data, the exploited software, servers, and materials were included in this published article. The datasets generated during and/or analyzed during the current study are available from the corresponding author on reasonable request.

## List of Abbreviations

DHA: Docosahexaenoic acid
DW: Dried weight
EPA: Eicosapentaenoic acid
FAME: Fatty acid methyl ester
MCCS: Microalgal culture collection of Shiraz university of medical sciences
PCR: Polymerase chain reaction
PUFA: Polyunsaturated fatty acid
SCP: Single-cell protein.

## Additional Points

Human and Animal Rights: this article does not contain any studies with human participants or animals performed by any of the authors.

## Supplementary Materials

Supplementary Materials Appendix A. Supplementary data: Supplementary data to this article can be found online.
