# Supplementary material for: North by Southwest: Screening the Naturally Isolated Microalgal Strains from Different Habitats of Iran for Various Pharmaceutical and Biotechnology Applications
Source: Int J Microbiol. 2022 Aug 12;2022:4386268. doi: 10.1155/2022/4386268 (PMC9391159; doi:10.1155/2022/4386268)
Supplement: Supplementary Materials — Appendix A. Supplementary data: Supplementary data to this article can be found online. [file 4386268.f1.docx]

Table S1: Morphological features of the selected strains.

| Strain | Figure | Main morphological features |
| --- | --- | --- |
| *Scenedesmus* sp. VN 002 | Fig. 3.1b | Single or 4-celled coenobia, egg-shape, chloroplast without pyrenoid, 3-5 µm diameter. |
| *Desmodesmus* sp. VN 004 | Fig. 3.1d | Single or 4-celled coenobia, cylindrical, single chloroplast with single pyrenoid, marginal spines (2 spines on each outer cells), asexual reproduction by autospores, 3-5 µm diameter. |
| *Desmodesmus* sp. VN 007 | Fig. 3.1g | Double row of 4-celled coenobia, spindle shape with acute cell poles, distinct pyrenoid, 3-5 µm diameter. |
| *Scenedesmus* sp. VN 001 | Fig. 3.1a | Single or 2-celled coenobia, spherical or spindle shape cells, parietal chloroplast with single pyrenoid, asexual reproduction by autospores, 3-5 µm diameter. |
| *Scenedesmus* sp. VN 003 | Fig. 3.1c | 2 or 4-celled coenobia, ellipsoidal with obtuse, single chloroplast with single big pyrenoid, marginal spines, 2 autospores released by rupture of parental cell wall (arrow) remaining visible in the colony, 3-5 µm diameter. |
| *Scenedesmus* sp. VN 005 | Fig. 3.1e | Coenobia, spindle shape, single chloroplast with single pyrenoid, asexual reproduction by autospores, 3-5 µm diameter. |
| *Scenedesmus* sp. VN 006 | Fig. 3.1f | Single or coenobia, fusiform cell with acute cell poles, 4-5 µm diameter. |
| *Scenedesmus* sp. VN 009 | Fig. 3.1i | 2 or 4-celled coenobia, spherical to ellipsoidal, single chloroplast with single big pyrenoid (sometimes parietal lens- shaped chloroplast or divided into several segments in mature cells), asexual reproduction by autospores, 4-5 µm diameter. |
| *Scenedesmus* sp. VN 010 | Fig. 3.1j | - |
| *Tetradesmus* sp. VN 008 | Fig. 3.1h | Single, 2 or 4-celled coenobia (joined along the longer axes), fusiform, single chloroplast with single pyrenoid, asexual reproduction by autospores, 4-5 µm diameter. |

Table S2: Growth parameters of investigated microalgal isolates.

| Code | Speciﬁc growth rate (d^-1^) | Doubling time (day) | Biomass yield (g L^-1^) | Biomass productivity (g L^-1^d^-1^) |
| --- | --- | --- | --- | --- |
| MCCS1 | 0.079 | 8.73 | 0.72 $\pm$ 0.03 | 0.040 $\pm$ 0.001 |
| MCCS2 | 0.070 | 9.86 | 0.50 $\pm$ 0.04 | 0.028 $\pm$ 0.002 |
| MCCS4 | 0.080 | 8.62 | 0.74 $\pm$ 0.09 | 0.040 $\pm$ 0.005 |
| MCCS5 | 0.081 | 8.52 | 0.50 $\pm$ 0.02 | 0.028 $\pm$ 0.001 |
| MCCS6 | 0.074 | 9.32 | 0.59 $\pm$ 0.03 | 0.033 $\pm$ 0.001 |
| MCCS13 | 0.072 | 9.58 | 0.53 $\pm$ 0.01 | 0.029 $\pm$ 0.000 |
| MCCS17 | 0.096 | 7.19 | 0.70 $\pm$ 0.07 | 0.039 $\pm$ 0.004 |
| MCCS18 | 0.066 | 10.45 | 0.59 $\pm$ 0.07 | 0.033 $\pm$ 0.004 |
| MCCS19 | 0.046 | 15 | 0.41 $\pm$ 0.06 | 0.023 $\pm$ 0.003 |
| MCCS20 | 0.072 | 9.58 | 0.63 $\pm$ 0.07 | 0.035 $\pm$ 0.004 |
| MCCS21 | 0.085 | 8.12 | 0.68 $\pm$ 0.07 | 0.038 $\pm$ 0.004 |
| MCCS22 | 0.097 | 7.11 | 0.43 $\pm$ 0.03 | 0.024 $\pm$ 0.001 |
| MCCS24 | 0.073 | 9.45 | 0.63 $\pm$ 0.08 | 0.035 $\pm$ 0.005 |
| MCCS25 | 0.073 | 9.45 | 0.79 $\pm$ 0.09 | 0.044 $\pm$ 0.005 |
| MCCS26 | 0.079 | 8.73 | 0.70 $\pm$ 0.06 | 0.039 $\pm$ 0.003 |
| MCCS27 | 0.068 | 10.15 | 0.58 $\pm$ 0.01 | 0.032 $\pm$ 0.000 |
| MCCS28 | 0.064 | 10.78 | 0.58 $\pm$ 0.09 | 0.032 $\pm$ 0.005 |
| MCCS29 | 0.082 | 8.41 | 0.71 $\pm$ 0.09 | 0.039 $\pm$ 0.005 |
| MCCS30 | 0.084 | 8.21 | 0.53 $\pm$ 0.09 | 0.029 $\pm$ 0.005 |
| MCCS31 | 0.055 | 12.54 | 0.39 $\pm$ 0.05 | 0.022 $\pm$ 0.003 |
| MCCS32 | 0.089 | 7.75 | 0.80 $\pm$ 0.10 | 0.044 $\pm$ 0.005 |
| MCCS33 | 0.113 | 6.11 | 0.73 $\pm$ 0.03 | 0.041 $\pm$ 0.002 |
| MCCS34 | 0.076 | 9.08 | 0.47 $\pm$ 0.04 | 0.026 $\pm$ 0.002 |
| MCCS35 | 0.085 | 8.12 | 0.97 $\pm$ 0.10 | 0.054 $\pm$ 0.005 |
| MCCS36 | 0.115 | 6.00 | 0.77 $\pm$ 0.05 | 0.043 $\pm$ 0.003 |
| MCCS37 | 0.086 | 8.02 | 0.30 $\pm$ 0.03 | 0.017 $\pm$ 0.002 |
| MCCS38 | 0.078 | 8.85 | 0.37 $\pm$ 0.06 | 0.021 $\pm$ 0.003 |
| MCCS39 | 0.086 | 8.02 | 0.34 $\pm$ 0.02 | 0.019 $\pm$ 0.001 |
| MCCS41 | 0.084 | 8.21 | 0.74 $\pm$ 0.08 | 0.041 $\pm$ 0.004 |

**
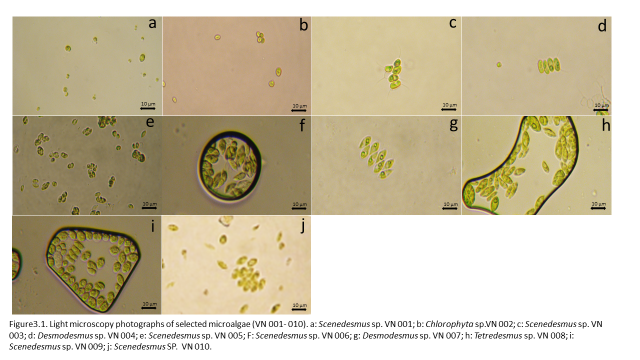
**

Figure S1: Light microscopy photographs of selected microalgae (VN 001- 010). a: *Scenedesmus* sp. VN 001; b: *Scenedesmus* sp.VN 002; c: *Scenedesmus* sp. VN 003; d: *Desmodesmus* sp. VN 004; e: *Scenedesmus* sp. VN 005; f: Scenedesmus sp. VN 006; g: *Desmodesmus* sp. VN 007; h: *Tetradesmus* sp. VN 008; i: *Scenedesmus* sp. VN 009; j: *Scenedesmus* SP. VN 010.
